# Quantitative Proteomics Analysis of FFPE Tumor Samples Reveals the Influences of NET-1 siRNA Nanoparticles and Sonodynamic Therapy on Tetraspanin Protein Involved in HCC

**DOI:** 10.3389/fmolb.2021.678444

**Published:** 2021-05-10

**Authors:** Bolin Wu, Haitao Shang, Jiayin Liu, Xitian Liang, Yanchi Yuan, Yichi Chen, Chunyue Wang, Hui Jing, Wen Cheng

**Affiliations:** ^1^ Department of Ultrasound, Harbin Medical University Cancer Hospital, Harbin, China; ^2^ Department of Interventional Ultrasound, Harbin Medical University Cancer Hospital, Harbin, China; ^3^ Institute of Cancer Prevention and Treatment, Heilongjiang Academy of Medical Science, Harbin Medical University, Harbin, China; ^4^ Department of Radiation Oncology, Harbin Medical University Cancer Hospital, Harbin, China

**Keywords:** hepatocellular carcinoma, sonodynamic therapy, tetraspanin protein, mass spectrometry, proteomics, ultrasound

## Abstract

Hepatocellular carcinoma (HCC) poses a severe threat to human health. The NET-1 protein has been proved to be strongly associated with HCC proliferation and metastasis in our previous study. Here, we established and validated the NET-1 siRNA nanoparticles system to conduct targeted gene therapy of HCC xenograft *in vivo* with the aid of sonodynamic therapy. Then, we conducted a label-free proteome mass spectrometry workflow to analyze formalin-fixed and paraffin-embedded HCC xenograft samples collected in this study. The result showed that 78 proteins were differentially expressed after NET-1 protein inhibited. Among them, the expression of 17 proteins upregulated and the expression of 61 proteins were significantly downregulated. Of the protein abundance, the vast majority of Gene Ontology enrichment terms belong to the biological process. The KEGG pathway enrichment analysis showed that the 78 differentially expressed proteins significantly enriched in 45 pathways. We concluded that the function of the NET-1 gene is not only to regulate HCC but also to participate in a variety of biochemical metabolic pathways in the human body. Furthermore, the protein–protein interaction analysis indicated that the interactions of differentially expressed proteins are incredibly sophisticated. All the protein–protein interactions happened after the NET-1 gene has been silenced. Finally, our study also provides a useful proposal for targeted therapy based on tetraspanin proteins to treat HCC, and further mechanism investigations are needed to reveal a more detailed mechanism of action for NET-1 protein regulation of HCC.

## Introduction

Worldwide, liver cancer is the fourth most common cause of cancer-related death and ranks sixth in terms of incident cases (Villanueva, 2019). With a 5-year survival of 18%, liver cancer is the second most lethal tumor after pancreatic cancer. Hepatocellular carcinoma is a major type of primary liver cancer. Changes in protein expression accompany HCC progress; thus, some proteins can be used as potential biomarkers for diagnosis and treatment (Ferrin et al., 2015).

In 1989, Prof. Yumita first reported sonodynamic therapy (SDT) (Yumita et al., 1989), which is based on the photodynamic therapy (PDT). This noninvasive treatment technology has already been known as a new anticancer strategy which uses nonthermal ultrasound energy in combination with sonosensitizer agents (Brown et al., 2004; Castano et al., 2004; Rosenthal et al., 2004; McHale et al., 2016). Normally, low-intensity focused ultrasound (LIFU, 1–3 MHz) frequency is used for this treatment technology to enhance the cavitation effect (Tachibana et al., 2008; Shibaguchi et al., 2011). The sonosensitizer agents together with ultrasound irradiation generate reactive oxygen species (ROS) that could induce cancer cell death under aerobic conditions (Yumita et al., 2010; Yumita et al., 2012).

Several reports have revealed that LIFU is able to enhance the anticancer effect of some chemotherapeutic drugs and improve cell membrane permeability. Our previous research studies have proved that the combination of LIFU irradiation and nanobubble system is regarded as an efficient and safe method for gene transfection (Wu et al., 2020; Wu et al., 2019). Besides, LIFU-combined NET-1 siRNA conjugated nanobubble system could effectively inhibit tumor growth and prolong the life of experimental animals (Shang et al., 2019).

Neuroepithelial transforming gene 1 (NET-1) is located at chromosome 10p15 and encodes a 54 kDa oncoprotein (Chan et al., 1996). It is a guanine nucleotide exchange factor involved in cytoskeletal regulation and cancer cell invasion (Murray et al., 2008). All NET genes have initially been identified as EST clones with sequences homologous to tetraspan, a superfamily which is distinguished by the presence of four transmembrane domains and has been implicated in signal transduction, cell adhesion, migration, proliferation, and differentiation (Serru et al., 2000; Kanetaka et al., 2001). NET-1 protein was known to be a member of the tetraspanin family (Ye et al., 2010). NET-1 protein has been identified in HCC, where it is a mediator of invasion and metastasis (Chen et al., 2007; Shen et al., 2008; Wu et al., 2013). Our previous research proved that the NET-1 protein had an impactful role in the proliferation and stiffness of HCC (Shang et al., 2019; Wu et al., 2019). Besides, the low expression of NET-1 protein also reduced the migration and invasive ability of HCC (Wu et al., 2016). However, the potential carcinogenic mechanism of NET-1 protein is still unclear.

Proteomics is an efficient research tool to reveal the mechanism and pathogeny of diseases on the proteinic level. Because of proteomics, analysis could analyze quite a lot of expressed proteins in tissues or cells; this revolutionary technology has been applied to identify HCC-related proteins in many studies (Chaerkady et al., 2008; Yang et al., 2017; Gao et al., 2019). However, altered expression of proteins quantified with conventional label-free proteomic methods was limited by the fresh or rapidly frozen tissue samples. Most human tumor samples archived in hospitals for pathologic diagnosis are formalin-fixed paraffin-embedded (FFPE), which have been widely used in the long-term preservation of tissues and organs (Bass et al., 2014). Besides, multifarious prescient technologies have been invented for transcriptomic (Li et al., 2014), genomic (Martelotto et al., 2017; Van Allen et al., 2014), proteomic, and protein (Mantsiou et al., 2019; Iglesias-Gato et al., 2016) from FFPE samples. In 1991, FFPE tissues have been analyzed for protein using antibodies as the invention of the heat-induced antigen retrieval (HIAR) technique for immunohistochemistry (IHC) (Shi et al., 1991). Afterward, kinds of different technologies have been applied to extract proteins from FFPE samples, which have extended the research of proteins to a proteomic level (Fowler et al., 2014; Wakabayashi et al., 2014; Broeckx et al., 2016). These studies have initially confirmed that FFPE samples can be used in mass spectrometry–based proteomic analysis.

Here, we established and validated the NET-1 siRNA nanoparticles system, which was then utilized for targeted gene delivery of HCC xenograft *in vivo* with the aid of SDT. Then, proteomic analyses of FFPE HCC xenograft samples were conducted to characterize the global quantitative protein expression profile and identify the differential protein expressions after gene therapy. Furthermore, we aimed to shed light on the functions of tetraspanin protein involved in HCC development and reveal the HCC-related proteins valuable for targeted therapy.

## Materials and Methods

### Preparation of Neuroepithelial Transforming Gene 1 siRNA Nanoparticles

The NET-1 siRNA duplex and the negative control duplex (NC-siRNA) were designed according to our previous research (Wu et al., 2019). The nanoparticles were prepared with DSPG, DSPC, and DSPE-PEG2000, and the weight ratio was 7:2:1. All the phospholipids were purchased from Avanti Polar Lipids (Avanti Polar Lipids, Alabaster, AL, United States). 20 mg of phospholipids were dissolved in a mixed solution of chloroform and methyl alcohol. The mixed solution was subsequently purged by vacuum rotary evaporation to form phospholipid thin film. Then, the thin film was hydrated at 40°C with 5 ml of DEPC-treated H_2_O. This was followed by dissolving the appropriate amounts of NET-1 siRNA duplex in the lipid film solution. The NET-1 siRNA nanoparticles and NC-siRNA duplex nanoparticles were obtained using an extrusion technology by mini-extruders (Avanti Polar Lipids, Alabaster, AL, United States) through a 400 nm membrane for 11 times. The obtained NET-1 siRNA nanoparticles and NC-siRNA duplex nanoparticles were then transferred into a sealed vial and stored at 4°C for further experiments.

The structure of NET-1 siRNA nanoparticles was detected under transmission electron microscope (TEM, Hitachi TEM system, Japan). The size and zeta potential were investigated by dynamic light scattering (DLS) via the Malvern Zetasizer Nanoseries (Zeta PALS BI-90 Plus, Brookhaven Instruments).

### Cell Lines and Animal Tumor Inoculation

The human HCC cell line HepG2 cells were a generous gift from the Institute of Cancer Research affiliated with Harbin Medical University (Harbin, China). Cells were cultured in DMEM medium supplemented with 10% fetal bovine serum and 1% penicillin/streptomycin in a humidified atmosphere containing 5% CO_2_/95% air at 37°C.

BALB/c nude female mice (6–8 weeks, 10–25 g) were purchased from Beijing Vital River Laboratory Animal Technology (Beijing, China). All the animals were housed in an environment with a temperature of 22 ± 1°C, a relative humidity of 50 ± 1%, and a light/dark cycle of 12:12 h. All animal studies (including the mice euthanasia procedure) were done in compliance with the regulations and guidelines of Harbin Medical University institutional animal care and conducted according to the Association for Assessment and Accreditation of Laboratory Animal Care and the Institutional Animal Care and Use Committee guidelines. Mice were anesthetized with 3% isoflurane inhalation and 1 L/min 100% oxygen. A total of 5 × 10^6^ HepG2 cells were suspended in 50 ml PBS and 50 ml Matrigel (BD Biosciences, San Jose, CA, United States). Tumor cell–Matrigel mixture (100 µl) was subcutaneously injected in the right back position of the mice.

### *In vivo* Studies

Once the tumor diameter reached 0.5 cm, the mice bearing tumors were randomly divided into five groups (six mice/group): group A, blank (PBS); group B, NET-1 siRNA nanoparticles without LIFU irradiation; group C, NET-1 siRNA nanoparticles with LIFU irradiation; group D, NC-siRNA duplex nanoparticles with LIFU irradiation; and group E, blank nanoparticles with LIFU irradiation. All the treatment nanoparticles were intravenously administered via the tail vein at a dose of 5 ml/kg body weight.

At 30 min postinjection, all groups were irradiated with LIFU using a CGZZ low-frequency ultrasound treatment instrument (Institute of Ultrasound Imaging, Second Affiliated Hospital of Chongqing Medical University, Chongqing, China). LIFU parameters were as follows: frequency of 1 MHz, pulse repetition frequency of 1 kHz, duty cycle yield of 50%, intensity of 1.0 W/cm^2^, and duration of irradiation of 5 min per mouse. The nude mice were treated twice a week for a total of 60 days. The survival end point was a tumor diameter of 20 mm in any direction (according to the guidelines for Tumor Induction in Mice and Rats, American Association for Laboratory Animal Science, Memphis, TN, United States); the maximum tumor diameter was measured twice every week using Aixplorer United States system with high-frequency probe (Super Linear TM SL15-4, Super Sonic Imagine, Aix-en-Provence, France). The HCC xenograft samples were harvested after euthanasia and formalin-fixed and paraffin-embedded for long-stem storage and further analyses.

### Immunohistochemical Staining

Half of the FFPE tumor samples were sectioned 4.5 μm thick and incubated 10 min with 0.3% H_2_O_2_ to block endogenous peroxidase. The sections were incubated overnight at 4°C with primary mouse antihuman NET-1 antibody (1:100, Abcam, Cambridge, United Kingdom) and then with secondary antibody at 37°C for 30 min. The sections were then stained with 3,3′-diaminobenzidine and were counterstained with hematoxylin, dehydrated in alcohol, and mounted. Quantitative analysis of total image staining was carried out by ImageJ software (v.1.80; National Institutes of Health, Bethesda, MD, United States).

### Proteomic Analysis

#### Total Protein Extraction

The rest of FFPE HCC xenograft samples in group A and group C were selected for proteomic analysis. The FFPE samples were dewaxed with octane and then hydrated with graded ethanol. After hydration, the sample was washed twice with PBS. After removing the PBS solution, an appropriate amount of protein lysate (4% SDS, 100 mM Tris, pH = 8.5) was added and incubated at 95°C for 10 min at room temperature, mixed by shaking and sonicated in an ice-water bath for 5 min. The samples were de-crosslinked with a refractive index at 95°C for 60 min and then reduced by adding an appropriate amount of TCEP and carboxyamidomethylated in CAA at 95°C for 5 min. The samples were sequentially centrifuged at 12,000*g* at 4°C for 15 min. Collecting the supernatant and adding four times the volume of precooling acetone at −20°C and precipitated it at −20°C for at least 4 h. Centrifuging at 12,000*g* for 15 min at 4°C. Collect the precipitate and air drying. An appropriate amount of protein solution (6 M urea, 100 mM TEAB, pH = 8.5) was added to dissolve the protein pellet.

#### Trypsin Treatment

The protein solution was added to flat membrane ultrafiltration (cut off molecular is 10 kDa) tube and was centrifuged at 14,000*g* at room temperature for 20 min, and the flow-through was discarded. 100 μl of 50 mM TEAB was added, and the sample was centrifuged at 14,000*g* at room temperature for 20 min. The washing procedure was repeated four times. 100 μl of 50 mM TEAB and an amount of 1:50 mass ratio of trypsin was added to the protein and incubated at 37°C overnight. After being centrifuged at 14,000*g* for 20 min, an equal volume of 2% formic acid was added. After mixing, the solution was centrifuged at 14,000*g* for 20 min at room temperature. The supernatant of flow-through was slowly passed through a C18 desalting column, and then, 1 ml washing solution (0.1% formic acid and 4% acetonitrile) was added to wash three times in succession, then 0.4 ml of eluent (0.1% formic acid and 75% acetonitrile) was added to elute twice in sequence, and the eluent samples were combined and freeze-dried.

### Liquid Chromatography–Mass Spectrometry/Mass Spectrometry Analysis

Mobile phase A (100% water and 0.1% formic acid) and B solution (80% acetonitrile and 0.1% formic acid) were prepared. The lyophilized powder was dissolved in 10 μl of solution A, centrifuged at 15,000 rpm for 20 min at 4°C, and 1 μg of the supernatant was injected into a homemade C18 Nano-Trap column (2 cm × 75 μm, 3 μm). Peptides were separated in a homemade analytical column (15 cm × 150 μm, 1.9 μm) using linear gradient elution, as listed in Supplementary Table S1. The isolated peptides were analyzed by the Q Exactive series mass spectrometer (Thermo Fisher), with ion source of Nanospray Flex™ (ESI), spray voltage of 2.3 kV, and ion transport capillary temperature of 320°C. Full scan ranges from m/z 350 to 1,500 with resolution of 60,000 (at m/z 200), an automatic gain control (AGC) target value was 3 × 10 6, and a maximum ion injection time was 20 ms. The top 20 (Huang et al., 2009) precursors of the highest abundant in the full scan were selected and fragmented by higher-energy collisional dissociation (HCD) and analyzed in MS/MS, where resolution was 15,000 (at m/z 200), the automatic gain control (AGC) target value was 5 × 10^4^, the maximum ion injection time was 45 ms, a normalized collision energy was set as 27%, and intensity threshold was 2.2 × 10^4^. The dynamic exclusion parameter was 20 s. The raw data of M.S. detection were named as “raw.”

### Data Analysis

#### The Identification and Quantitation of Protein

The resulting spectra from each fraction were searched separately against the Homo sapiens UniProt database by the search engines: Proteome Discoverer 2.2 (PD 2.2, Thermo). The search parameters are set as follows: mass tolerance for precursor ion was ten ppm and mass tolerance for production was 0.02 Da. Carbamidomethyl was specified in PD 2.2 as fixed modifications. Oxidation of methionine (M) and acetylation of the N-terminus was specified in PD 2.2 as variable modifications. A maximum of 2 missed cleavage sites was allowed.

The identified protein contains at least one unique peptide with FDR no more than 1.0%. Proteins containing similar peptides that could not be distinguished by MS/MS analysis were identified as the same protein group. Precursor ion was quantified by a label-free quantification method based on intensity. Mann–Whitney Test statistically analyzed the protein quantitation results for proteins whose quantitation significantly different between experimental and control groups were defined as differentially expressed proteins (DEP).

#### The Functional Analysis of Protein and Differentially Expressed Proteins

Gene Ontology (GO) and InterPro (IPR) analysis were conducted using the InterProScan-5 program against the nonredundant protein database (including Pfam, PRINTS, ProDom, SMART, ProSiteProfiles, and PANTHER) (Jones et al., 2014); the databases of Clusters of Orthologous Groups (COG) and Kyoto Encyclopedia of Genes and Genomes (KEGG) were used to analyze the protein family and pathway. The probable protein–protein interactions (PPI) were predicted using the STRING-db server (Franceschini et al., 2013) (http://string.embl.de/). The enrichment pipeline was used for enrichment analysis of G.O, IPR, and KEGG (Huang et al., 2009).

### Statistical Analysis

All data are presented as mean ± standard error of the mean (SEM). Statistical analysis was performed by unpaired, two-tailed Students t-test using the GraphPad Prism 8.0 software (GraphPad Software) if not denoted otherwise. Differences were considered statistically significant at *p*-value < 0.05. The Mann–Whitney test statistically analyzed the protein quantitation results. Because >two groups were compared in this survival study, the log-rank test with Bonferroni correction was used to compare two specific groups when the overall values were *p* < 0.05. Proteins were supposed to be significantly differentially expressed when the *p*-value < 0.05, fold change (FC) ≤ 0.05 or *p*-value < 0.05, FC ≥ 2.0.

## Results

### Characterization of Neuroepithelial Transforming Gene 1 siRNA Nanoparticles

**FIGURE 1:**
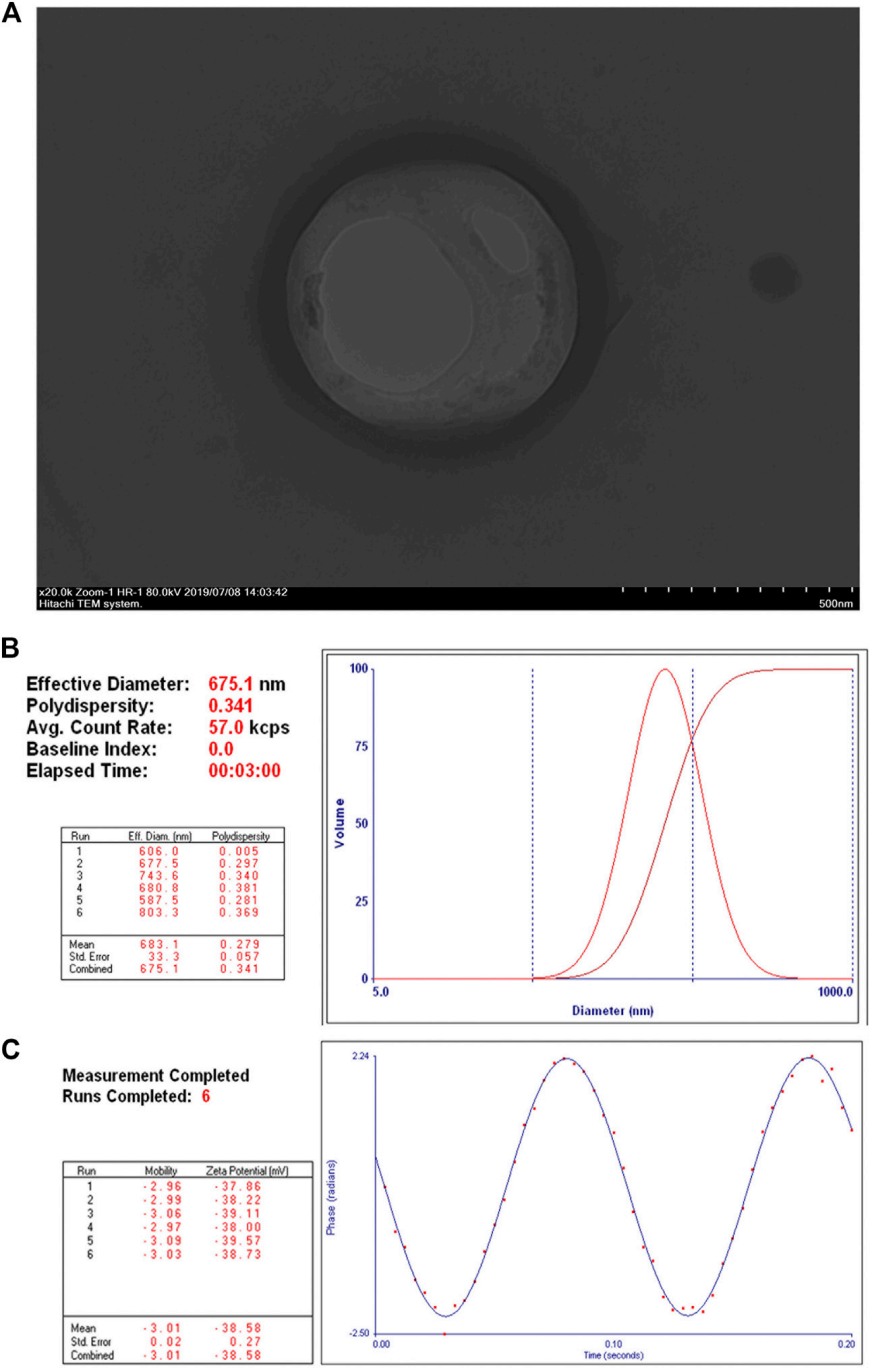
Structure and characterization of NET-1 siRNA nanoparticles. **(A)** Transmission electron microscopy image showed a quasi-spherical morphology of NET-1 siRNA nanoparticles with a diameter of about 600 nm. Original magnification, ×20,000. Scale bar, 500 nm; **(B)** the DLS results showed a mean particle size of NET-1 siRNA nanoparticles to be 675.1 ± 33.3 nm with a 0.341 polydispersity value; **(C)** the Zeta PALS BI-90 Plus analyzer indicated a surface zeta potential of NET-1 siRNA nanoparticles to be –38.58 ± 0.27 mV.

The obtained NET-1 siRNA nanoparticles were well dispersed in aqueous solution and appeared as quasi-spheres with nanosize by transmission electron microscopy observation (Figure 1A). The mean particle size of NET-1 siRNA nanoparticles was 675.1 ± 33.3 nm, with a 0.341 mean polydispersity index (Figure 1B). Meanwhile, the zeta potential value of the complexes was –38.58 ± 0.27 mV (Figure 1C).

### Analysis of *In Vivo* Study

**FIGURE 2:**
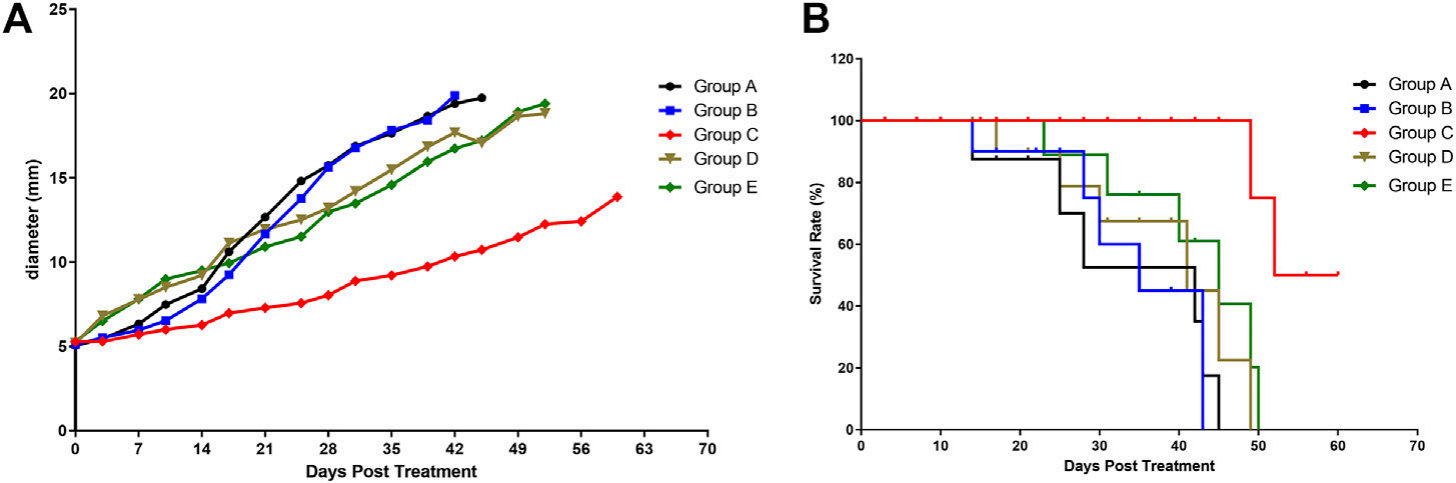
Tumor growth and mice survival in different mice groups. **(A)** Effect of the various treatments on tumor growth. **(B)** Effect of the various treatments on follow-up survival of mice (*n* = 6/group). Group A, blank control (black curve), group B, NET-1 siRNA nanoparticles without LIFU irradiation (blue curve); group C, NET-1 siRNA nanoparticles with LIFU irradiation (red curve); group D, NC-siRNA duplex nanoparticles with LIFU irradiation (brown curve); and group E, blank nanoparticles with LIFU irradiation (green curve).

A decrease in tumor growth was observed in group C compared with other groups (*p* = 0.0461, Figure 2A). During the 60 days follow-up, all mice with a tumor larger than 2 cm were euthanized (according to the guidelines for Tumor Induction in Mice and Rats).

Briefly, of group A, three mice died a natural death and three mice were euthanized within 45 days, the median survival was 28 days (Figure 2B, black). Of group B, 4 mice died a natural death within 35 days (Figure 2B, blue), and the other 2 mice were euthanized owing to the tumor size (Figure 2A, blue). Of group D, all mice died within 49 days (Figure 2B, brown). Of group E, all mice died within 50 days (Figure 2B, green).

Conversely, the group C showed the best survival result. 4 mice in group C survived until the end of follow-up (Figure 2B, red), and none of the tumors on any of the mice exceeded 20 mm in any direction during the follow-up period (Figure 2A, red). The log-rank (Mantel–Cox) test showed that the survival curves were significantly different (*p* = 0.0043).

### Analysis of Immunohistochemical Staining

**FIGURE 3:**
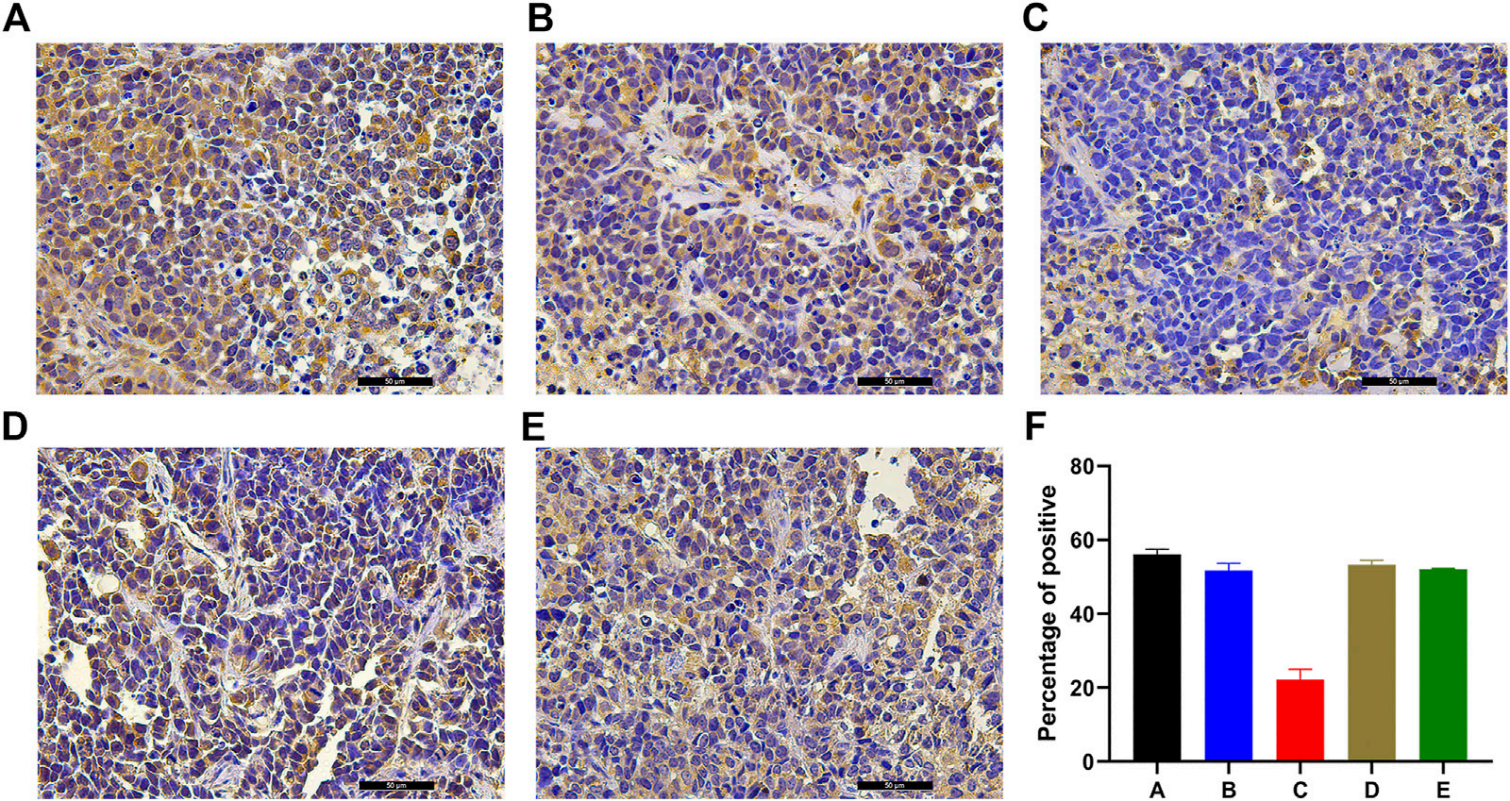
Example of the variability of IHC staining within tumor samples. **(A)** Group A showed strong staining, indicating the HCC cells surrounded by NET-1 protein (brown). **(B)** There was also an amount of NET-1 proteins around the tumor cells in group B. **(C)** There was little brown staining around the tumor, which indicated the NET-1 protein was significantly inhibited in group C. **(D**, **E)** There were also a large number of NET-1 proteins around tumor cells in group D and group E. **(F)** Quantitative IHC analysis of all groups, original magnification, ×400, Scale bar, 50 µm.

Massive positive immunostaining for NET-1 protein was observed in group A, group B, group D, and group E tissues (Figures 3A–E). However, the NET-1 protein expression was significantly downregulated in groups C compared with the other groups (Figure 3C).

The percentage of positive cells was quantified by ImageJ. Quantitative IHC analysis indicated that the NET-1 protein staining was 56.11 ± 1.37 (group A), 51.69 ± 2.01 (group B), 22.24 ± 2.81 (group C), 53.29 ± 1.23 (group D), and 52.07 ± 0.24 (group E) compared with the whole picture in each group (Figure 3F, *p* < 0.01, one-way ANOVA).

### Analysis of Protein Abundance

**FIGURE 4:**
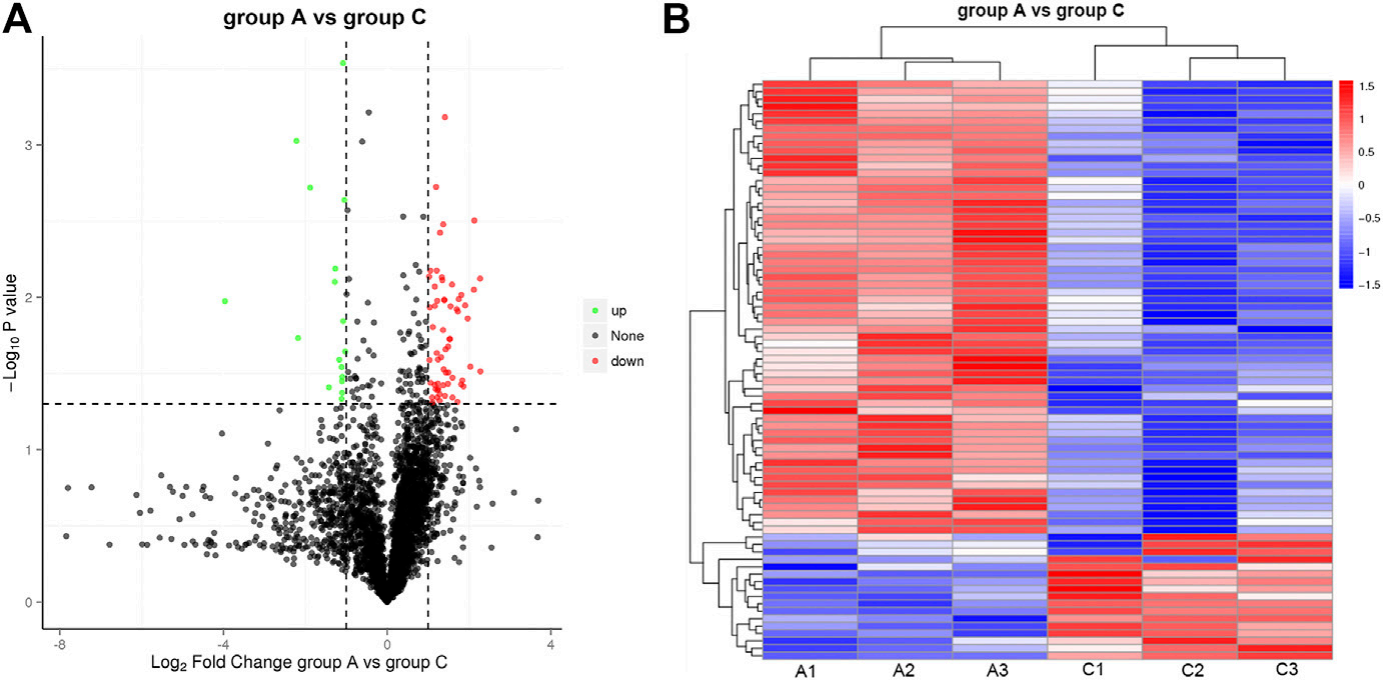
Illustration of DEP quantified from the label-free analysis **(A)** Volcano plots of protein abundance between group A and group C. Green dots represent downregulated protein (FC ≤ 0.05, *p*-value≤0.05), and red dots represent upregulated protein (FC ≥ 2.0, *p*-value≤0.05). **(B)** Heat map of hierarchical clustering of DEP. Red indicates high expression protein, and blue indicates low expression protein.

Inspired by the IHC results, we carried out a label-free proteome analysis using FFPE HCC xenograft samples. In total, 3,389 proteins were quantified from the label-free analysis. Compared to Group A, a total of 78 proteins were differentially expressed (*p*-value ≤ 0.05, Figure 4A). Among them, the expression of 17 proteins were significantly upregulated (FC ≥ 2.0, *p*-value ≤ 0.05) and the manifestation of 61 proteins were significantly downregulated (FC ≤ 0.05, *p*-value ≤ 0.05). Cluster analysis of protein abundance was shown by a heat map (Figure 4B). Red indicates high expression proteins and blue indicates low expression proteins.

### Analysis of Gene Ontology Functional Enrichment

**FIGURE 5:**
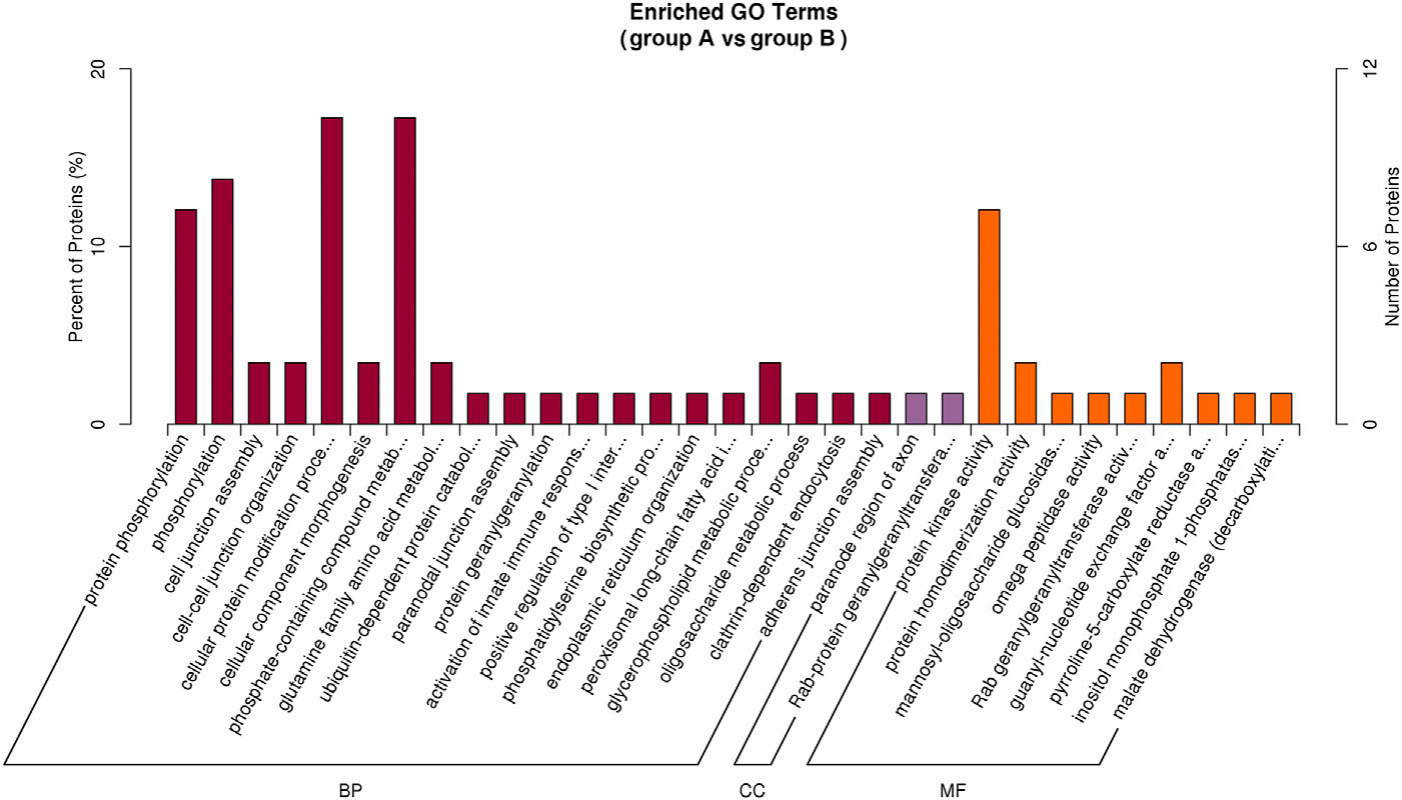
Illustration of the top 20 G.O. enrichment terms of all DEP. There are three categories of G..O. enrichment terms, biological process (B.P.), molecular function (M.F.), and cellular component (CC). The most DEP enriched in biological process (dark red), and only two CC terms had DEP enriched (purple). There were nine terms of MF (orange) were enriched by DEP.

Based on the G.O. enrichment analysis, we can explore the main biological functions of DEP. The representative five enriched G.O. details of DEP were shown in Table 1. The top 20 G.O. enrichment terms of DEP were illustrated in Figure 5. The vast majority of G.O. enrichment terms belong to the biological process (B.P.). The enriched G.O. terms of the biological process were exhibited in detail in directed acyclic graph (DAG), as shown in Supplementary Figure S1A. Besides, the enriched G.O. terms of molecular function (M.F.) and cellular component (CC) were shown in Supplementary Figure S1B,C, respectively. The complete result of G.O. enrichment analysis was shown in Supplementary Table S2. The top 10 G.O. enrichment terms were presented graphically by DAG (Supplementary Figure S1).

**TABLE 1:** Representative five enriched G.O. terms.

G.O. ID	G.O. term	G.O. class	*p* value	Protein ID
G.O.:0006468	Protein phosphorylation	B.P.	0.001063999	Q00534, P27361, F8W6G1, A0A024QZY5, O14976, B4E2L0, P12931
G.O.:0004672	Protein kinase activity	M.F.	0.001220283	Q00534, P27361, F8W6G1, A0A024QZY5 O14976, B4E2L0, P12931
G.O.:0034329	Cell junction assembly	B.P.	0.002098766	A0A2R8Y5A3, P78357
G.O.:0042803	Protein homodimerization activity	M.F.	0.003455028	B7Z9B1, B4DL07
G.O.:0033270	Paranode region of axon	CC	0.019097794	P78357

CC represents cellular component, M.F. represents molecular function, B.P. represents biological process.

### Analysis of Kyoto Encyclopedia of Genes and Genomes Pathway Enrichment

**FIGURE 6:**
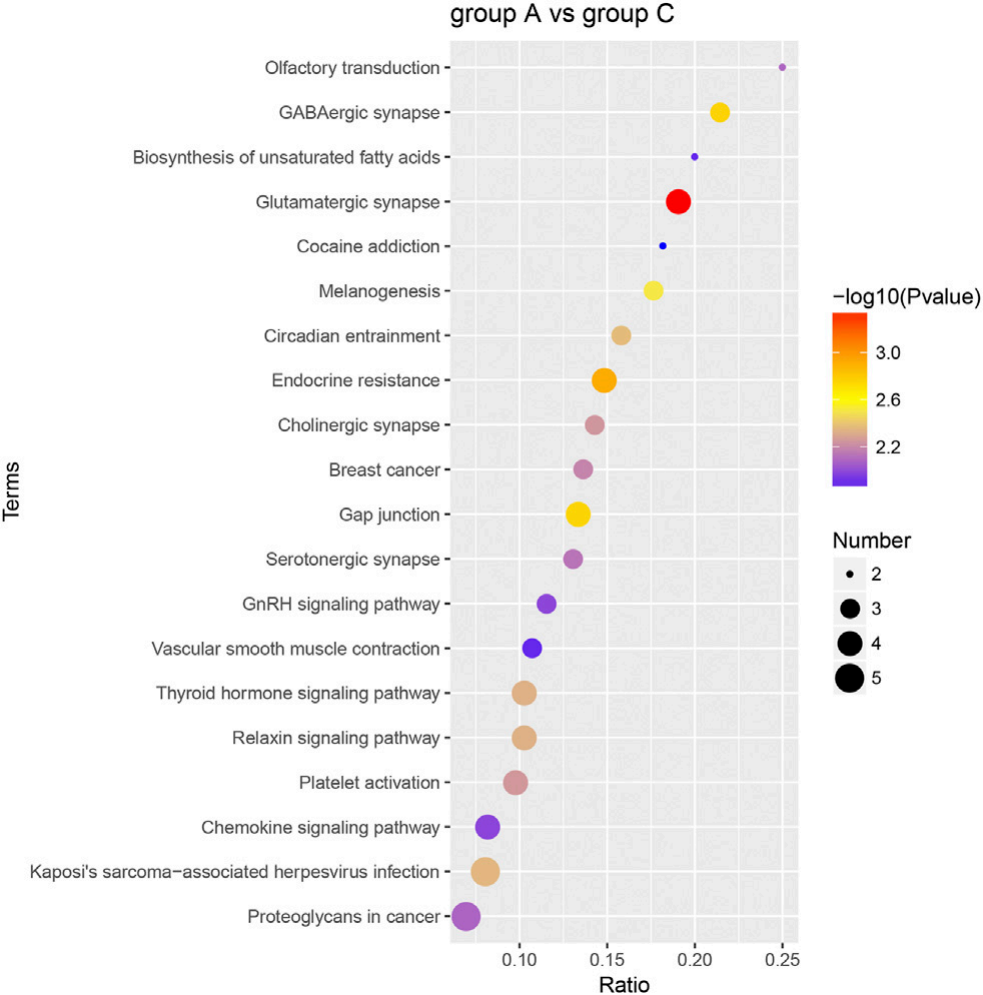
KEGG pathway enrichment analysis of DEP. The top 20 KEGG enrichment terms of all DEP between group A and group C were shown in the scatter plot. The color of the dot represents the −log10 (*p*-value), and the size of the dot represents the number of DEP.

The dominant biochemical metabolic pathways and signal transduction pathways, which were regulated by DEP, could be identified by KEGG pathway enrichment analysis. The representative five enriched KEGG pathways of DEP were “glutamatergic synapse,” “endocrine resistance,” “GABAergic synapse,” “gap junction,” and “melanogenesis” (Table 2). The top 20 enriched KEGG pathway terms were presented by a scatter plot (Figure 6). The details of all KEGG pathway enrichment analysis were shown in Supplementary Table S3.

**TABLE 2:** Representative five enriched KEGG pathway terms of DEP.

Map ID	Map title	*p* value	Protein ID	Description
map04724	Glutamatergic synapse	0.000434861	A0A024R056, P27361, B4E2L0, Q5U0L9	Guanine nucleotide binding protein (G protein), beta polypeptide 1, isoform CRA a, mitogen-activated protein kinase 3, cDNA FLJ54730, highly similar to cAMP-dependent protein kinase, beta-2-catalytic subunit, homer homolog 3 (Drosophila)
map01522	Endocrine resistance	0.001179526	P27361, P42773 B4E2L0, P12931	Mitogen-activated protein kinase 3,Cyclin-dependent kinase 4 inhibitor C, cDNA FLJ54730, highly similar to cAMP-dependent protein kinase, beta-2-catalytic subunit, proto-oncogene tyrosine-protein kinase Src
map04727	GABAergic synapse	0.001725647	A0A024R056, B4E2L0 P12931	Guanine nucleotide binding protein (G protein), beta polypeptide 1, isoform CRA a, cDNA FLJ54730, highly similar to cAMP-dependent protein kinase, beta-2-catalytic subunit, proto-oncogene tyrosine-protein kinase Src
map04540	Gap junction	0.001771527	P04350, P27361 B4E2L0, P12931	Tubulin beta-4A chain, mitogen-activated protein kinase 3, cDNA FLJ54730, highly similar to cAMP-dependent protein kinase, beta-2-catalytic subunit, proto-oncogene tyrosine-protein kinase Src
map04916	Melanogenesis	0.003105093	A0A2R8Y5A3, P27361 B4E2L0	Catenin beta-1, mitogen-activated protein kinase 3, cDNA FLJ54730, highly similar to cAMP-dependent protein kinase, beta-2-catalytic subunit

### Analysis of InterPro Enrichment

**FIGURE 7:**
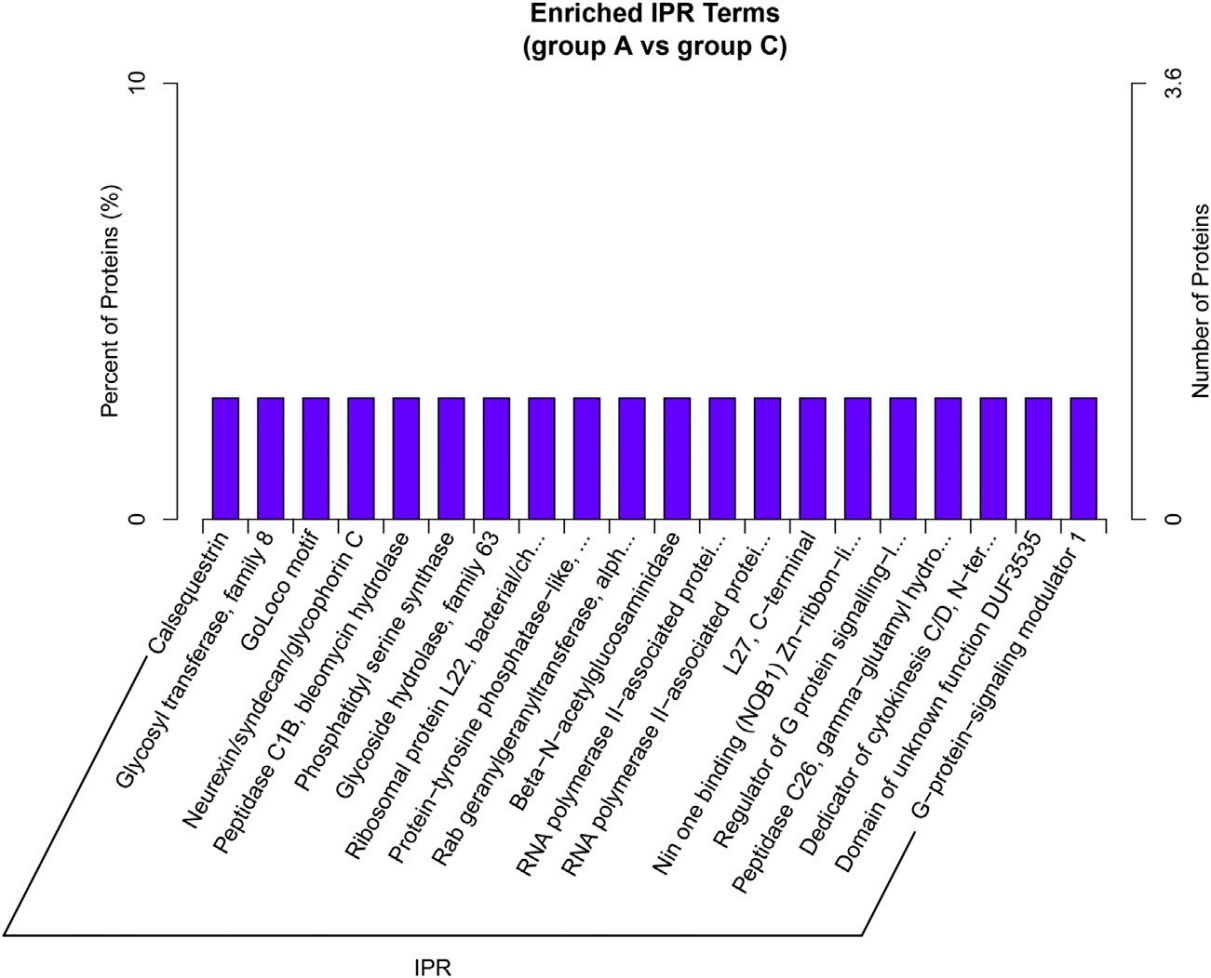
IPR enrichment analysis. A total of 47 IPR terms were identified between group A and group C. The top 20 IPR enrichment terms of all DEP were shown in the bar plot. Each IPR terms has only one DEP enriched.

In this study, we identified 47 IPR terms with differential enrichment (Supplementary Table S4). The representative five enriched IPR IDs and titles of DEP were shown in Table 3. The top 20 enriched IPR terms were explained in Figure 7. The details of all IPR enrichment terms were explained in Supplementary Table S4.

**TABLE 3:** Representative five enriched IPR terms of DEP.

IPR ID	IPR title	*p* value	Protein ID	Description
IPR001393	Calsequestrin	0.012354152	P31415	Calsequestrin-1
IPR002495	Glycosyl transferase, family 8	0.012354152	B2R5R5	cDNA, FLJ92583, highly similar to homo sapiens glycogenin (GYG), mRNA
IPR003109	GoLoco motif	0.012354152	A0A0A0MRC4	G-protein-signaling modulator 1
IPR003585	Neurexin/syndecan/glycophorin C	0.012354152	P78357	Contactin-associated protein 1
IPR004134	Peptidase C1B, bleomycin hydrolase	0.012354152	Q13867	Bleomycin hydrolase

### Protein–Protein Interaction Analysis

**FIGURE 8:**
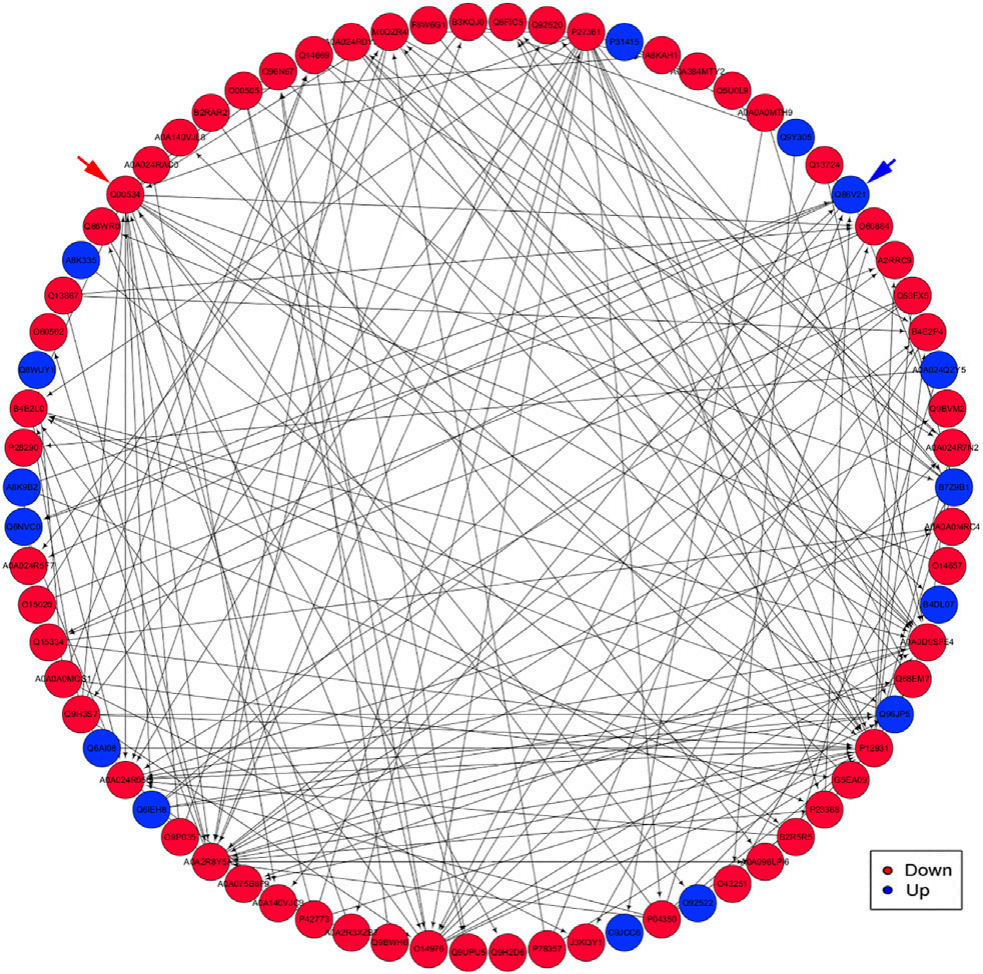
An interaction network of PPI analysis. The network revealed the direct and potential regulatory relationships between DEP. The downregulated proteins are represented by red nodes. The upregulated proteins are represented by blue nodes. Blue arrow indicates the Q86V21 (AACS) protein. Red arrow indicates the Q00534 (cyclin-dependent kinase 6) protein.

PPI analysis indicated that the significantly upregulated proteins P12931, A0A2R8Y5A3, Q00534, and P27361 and the significant downregulated proteins P31415, A0A024QZY5, B7Z9B1, B4DL07, and A8K335 were interrelated and interacted with each other (Figure 8).

## Discussion

In our previous study, the NET-1 protein functions of HCC were investigated *in vivo* (Wu et al., 2019). The expression of NET-1 protein in HCC xenograft was merely detected by IHC staining. In the present study, we hypothesized that the combination of NET-1 siRNA nanoparticles system and SDT could effectively inhibit the expression of NET-1 protein and regulate multiple proteins. Compared with preexisting combined anticancer strategies, this established strategy of nanoparticles system integrated with SDT offers distinct advantages, such as high penetration, noninvasive, and flexible choice of tumor site. The obtained nanoparticles could be employed as gene vehicle and cavitation nuclei for acoustic cavitation effect with SDT, which could dig holes of approximately 300 nm on the tumor cell membranes (Meng et al., 2019). The acoustic cavitation effect has minimal harmful influences on adjacent tissues or organs and is depicted to be a potential strategy for gene delivery (Miansari et al., 2019). Also, as evidenced by IHC staining, the expression of NET-1 protein in group C was significantly depressed (Figure 3).

Label-free proteome analysis was conducted to identify the protein abundance. Surprisingly, a total of 78 DEP were sifted from 3,389 quantified proteins (*p*-value≤0.05). Moreover, 17 proteins were significantly upregulated (green dots in Figure 4A, FC ≥ 2.0, *p*-value ≤ 0.05), such as Q86V21, which was described as acetoacetyl-CoA synthetase (AACS). AACS is highly expressed in the brain. It is a ketone body-utilizing enzyme for the synthesis of cholesterol and fatty acids, which are essential, abundant components of neuronal tissue. It has been proved that AACS is regulated by SREBP-2 and involves in the normal development of neurons (Hasegawa et al., 2012). In contrast, 61 proteins were significantly downregulated (red dots in Figure 4A, FC ≤ 0.05, *p*-value ≤ 0.05), such as Q00534, which was described as cyclin-dependent kinase 6 (CDK6). Amplification of CDK6 and overexpression of cyclin D protein are also frequent in human cancers (Beroukhim et al., 2010). The expression of CDK6 can be downregulated as a result of NET-1 protein depression, which provides a therapeutic potential for targeting CDK6 in the treatment of cancer. Our hypothesis was confirmed preliminarily. Encouraged by the proof-of-concept results, we analyzed the potential functions of DEP based on the mass spectra proteome analysis results.

First, G.O. enrichment analysis for biological process, cell component, and molecular function was performed. A total of 78 G.O. enrichment terms belong to the biological process, which would indicate that most of the DEP was involved in regulating the biological process of HCC. On the other hand, NET-1 protein was a member of the tetraspanin family, which was is a crucial point of HCC biological processes such as proliferation, differentiation, migration, and invasion (Mazzocca et al., 2008; Mazzocca et al., 2014). Consequently, silencing the NET-1 gene could regulate many HCC biological processes by depressing the NET-1 protein expression. Second, KEGG pathway enrichment analysis was performed to explore the pathways, which may be regulated by the NET-1 gene. A total of 45 significantly enriched pathways were identified (*p*-value ≤ 0.05), as shown in Supplementary Table S3. In organisms, different proteins coordinate their biological behaviors, and the pathway-based analysis helps to understand their biological functions further. Interestingly, the DEP enriched in multifarious pathways, not only cancer-related pathways but also many other pathways, such as “olfactory transduction” (map04740), “cocaine addiction” (map05030), “morphine addiction” (map05032), “Kaposi’s sarcoma-associated herpesvirus infection” (map05167), and “human papillomavirus infection” (map05165). We concluded that the function of the NET-1 gene is not only to regulate HCC but also to participate in a variety of biochemical metabolic pathways in the human body. Third, proteins are composed of IPR that are units of protein structure, function, and evolution. The study of the IPR of proteins is essential for understanding the biological role of proteins and their development. In this study, 47 IPR enrichment terms were found, as shown in Supplementary Table S4 (*p*-value ≤ 0.05). Each IPR had a specific protein corresponding to it. It could be proved that IPR can form new proteins by copying and combining. The combination distribution between different IPR does not conform to the random model but shows that some IPR has a powerful combination ability, some of which are rarely combined with other domains. Finally, PPI analysis revealed the direct and potential regulatory relationships between DEP. As shown in Figure 8, all DEP formed a big circle and each node represented a DEP. The upregulated proteins are represented by blue nodes and the downregulated proteins are represented by red nodes. The interactions of DEP are incredibly sophisticated. All the protein–protein interactions happened after the NET-1 gene has been silenced. For instance, the Q86V21 (AACS) protein (blue arrow in Figure 8) could regulate the A0A024R5F7 protein, which was described as 7-dehydrocholesterol reductase isoform 1. It also indirectly proves that NET-1 protein could regulate the synthesis of cholesterol by modulating AACS and 7-dehydrocholesterol reductase isoform 1. The Q00534 (cyclin-dependent kinase 6) protein (red arrow in Figure 8) could regulate the other 11 proteins, such as O60884 (DNAJA2), O14976 (GAK) and so on. GAK is a cellular serine/threonine kinase that plays a major role in clathrin-mediated membrane trafficking (Wouters et al., 2019). It has been proved that osteosarcoma cell proliferation and survival are dependent on GAK (Susa et al., 2010). Thus, the NET-1 protein may be a potential therapeutic target of osteosarcoma.

In summary, for the first time, our present study provides valuable insight into the regulation of NET-1 siRNA nanoparticles system and SDT on other proteins in HCC on a proteomics level. It proved that the NET-1 protein, one of the tetraspanin proteins, participated in regulating many critical signaling pathways in HCC development. Our results also provide a potential proposal for targeted therapy based on tetraspanin proteins to treat HCC, and further mechanism investigations are needed to reveal a more detailed mechanism of action for NET-1 protein regulation of HCC.

## Data Availability

The mass spectrometry proteomics data have been deposited to the ProteomeXchange Consortium via the PRIDE partner repository with the dataset identifier PXD020763.
