# Supplementary material for: Quantitative Proteomics Analysis of FFPE Tumor Samples Reveals the Influences of NET-1 siRNA Nanoparticles and Sonodynamic Therapy on Tetraspanin Protein Involved in HCC
Source: Front Mol Biosci. 2021 May 10;8:678444. doi: 10.3389/fmolb.2021.678444 (PMC8141748; doi:10.3389/fmolb.2021.678444)
Supplement: Supplementary file 3 [file Table1.DOCX]

| Time  (min) | flow rate  (nL/min) | mobile phase A  (%) | mobile phase B  (%) |
| --- | --- | --- | --- |
| 0 | 600 | 94 | 6 |
| 2 | 600 | 90 | 10 |
| 49 | 600 | 70 | 30 |
| 52 | 600 | 65 | 35 |
| 54 | 600 | 50 | 50 |
| 55 | 600 | 0 | 100 |
| 60 | 600 | 0 | 100 |

Supplementary Table 1 Liquid chromatography elution gradient table
