# Supplementary figures and images for: Quantitative Proteomics Analysis of FFPE Tumor Samples Reveals the Influences of NET-1 siRNA Nanoparticles and Sonodynamic Therapy on Tetraspanin Protein Involved in HCC
Source: Front Mol Biosci. 2021 May 10;8:678444. doi: 10.3389/fmolb.2021.678444 (PMC8141748; doi:10.3389/fmolb.2021.678444)

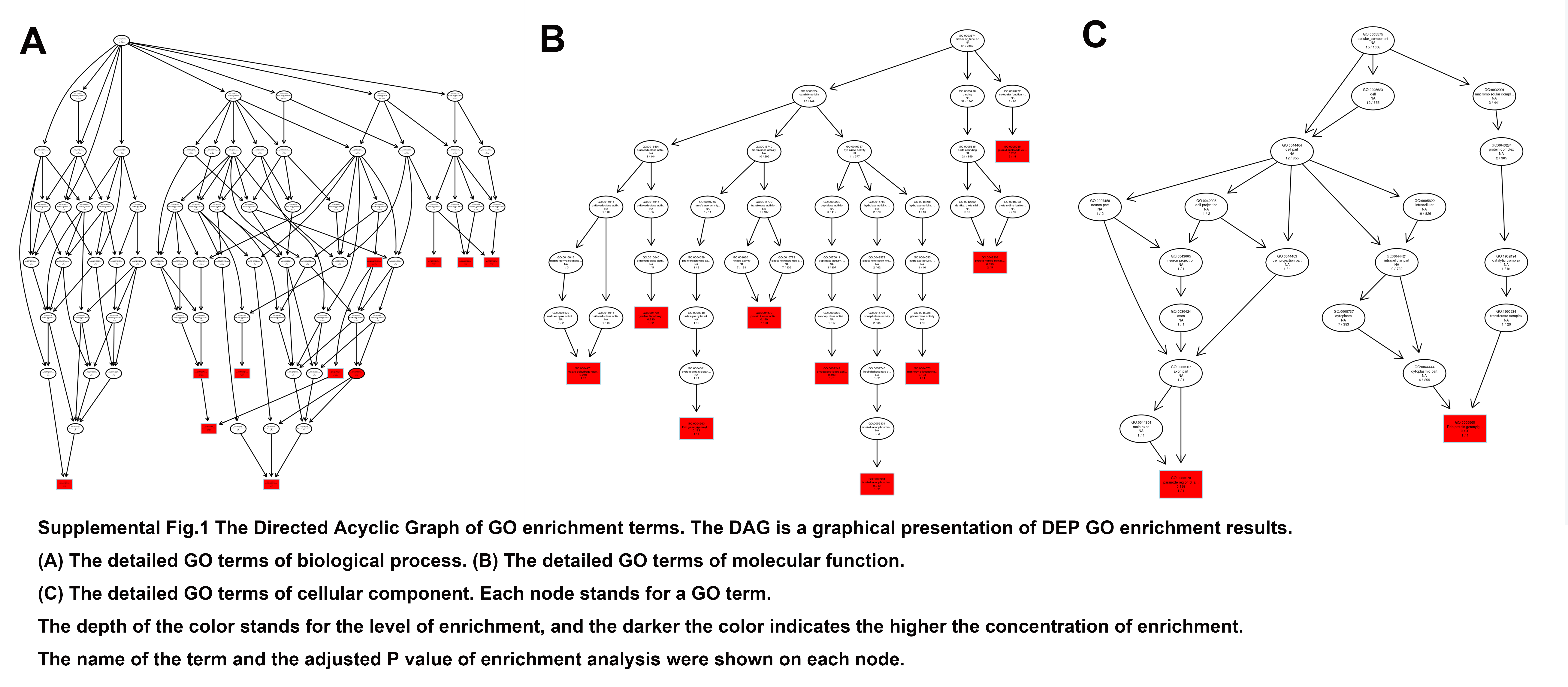

Supplement: Supplementary file 5 [file Image1.TIF]
